# Self-Reported Eating Speed Is Associated with Indicators of Obesity in Adults: A Systematic Review and Meta-Analysis

**DOI:** 10.3390/healthcare9111559

**Published:** 2021-11-16

**Authors:** Ezgi Kolay, Aleksandra Bykowska-Derda, Safa Abdulsamad, Malgorzata Kaluzna, Karolina Samarzewska, Marek Ruchala, Magdalena Czlapka-Matyasik

**Affiliations:** 1 Department of Human Nutrition and Dietetics, Poznan University of Life Sciences, 31 Wojska Polskiego St., 60-624 Poznan, Poland; ezgi.kolay@up.poznan.pl (E.K.); aleksandra.derda@up.poznan.pl (A.B.-D.); safaabdulsamad@yahoo.com (S.A.); 2 Department of Endocrinology, Metabolism and Internal Medicine, Poznan University of Medical Sciences, 49 Przybyszewskiego St., 60-355 Poznan, Poland; mkaluzna@ump.edu.pl (M.K.); mruchala@ump.edu.pl (M.R.); 3 Department of Clinical Auxiology and Pediatric Nursing, Poznan University of Medical Sciences, 27/33 Szpitalna, 60-572 Poznan, Poland; ksamarzewska@ump.edu.pl

**Keywords:** speed eating, anthropometry, obesity, adults, waist circumference, body mass index

## Abstract

Eating speed (ES) as a dietary behaviour has become a widely discussed factor for weight management and obesity. This study analysed the relationship between ES and anthropometric indicators of obesity, including BMI and waist circumference (WC) in adults. A search conducted of PubMed, Web of Science, Science Direct and Scopus found six longitudinal studies and fifteen cross-sectional studies published for further analysis. A quality assessment was performed with the MINORS checklist. Eight studies were included in the meta-analysis and almost all reviewed studies showed that ES was associated with BMI, and non-fast eaters had significantly lower BMI than fast eaters. Therefore, it was assumed that slowing down the ES may be an effective strategy for weight management and lowering obesity risk. There was also an association between WC and ES. Assessment of eating speed can be included in nutrition surveys to analyse obesity risk. More broadly, research is also needed to establish a validated and standardised methodology to determine eating speed. Further research needs to examine the links between eating speed, obesity, ethnicity, sex, food culture and chronic diseases.

## 1. Introduction

According to the WHO, obesity is continuously growing worldwide and has tripled in the last forty years [1]. Its relationship with noninfectious diseases, e.g., diabetes, hypertension, dyslipidaemia and metabolic syndrome, reducing life expectancy and quality of life, are well known [2,3]. The revealed obesity-related markers presented in the literature have been widely reported. Among the mentioned markers, a sedentary lifestyle [4], socioeconomic factors [5], genetics [6], microbiome [7], psychological well-being [2], endocrine regulation [3], family and education influence [8,9], dietary behaviours such as skipping breakfast [10], snacking [11] and meal regulations [12,13,14] are prominent. Despite confirmed associations between obesity and its markers, there is a constant search for (and analysis of) new dependencies to manage and slow the 21st-century epidemic. As the most critical factor in obesity, dietary behaviour plays a crucial role in weight management and prevention of obesity through its influence on energy balance and close interaction with other factors, such as nutrigenomics and psychology [15,16].

The perception of eating behaviours has evolved and extended its scope of understanding in recent years. Dietary behaviours and food choices have been extensively investigated in recent years [17,18]. They are defined as the broad spectrum of dietary lifestyle-related modern consumer behaviours, including hours of meal intake, shortening the time to prepare meals, food preparation methods and eating speed [19,20]. The speed of information flow and decision making have gained importance in nutrition [21,22]. These aspects are related to eating speed, as one of the elements accompanying modern consumer life under time pressure. Therefore, customers worldwide choose easy-to-prepare meals, which are often highly processed, and spend long hours in front of screens both at work and in their free time. Along with this change in the eating behaviours of the contemporary consumer, the terms ‘eating speed’ and ‘eating rate’ began to be used. Eating speed is defined as the length of time of eating; in contrast, the eating rate is determined by the total food consumption in grams per minute or kcal per minute, specified for certain foods [23,24]. Eating rate and eating speed have often been used interchangeably in the literature [25,26,27,28]. This terminological confusion can lead to classification errors and make meta-analysis studies challenging to perform. Eating speed is a simple tool that can be used in cohort studies, but unlike the eating rate, it does not require equipment, a laboratory visit, or financial resources—making it suitable for use in large populations as a predictor and marker of obesity.

In this context, previous studies have shown that slow consumption is related to lower energy intake. They also indicated the importance of slowing down eating as a necessary factor in improving dietary habits [29]. However, the relations of eating speed, satiety and obesity are still discussed and explained differently. Firstly, the mechanism is considered a hormone-independent factor influencing body weight by decreasing energy intake and body weight [30,31]. Secondly, hormonal influences were also noted. Ghrelin, peptide YY and glucagon-like peptide-1 (GLP-1) are known as gastrointestinal hormones that significantly affect hunger and satiety [32]. A randomised control trial investigated the effect of eating speed on these hormones and found that consuming the same food more slowly increased the peptide YY and GLP-1 concentrations [33]. The relations between sex hormones affecting neurotransmitters and hunger-satiety hormones, energy intake and weight management were discussed [34,35]. Therefore, slowing down eating might be related to lower incidences of obesity [36,37]. Independently of the mechanism postulated in literature, many experimental studies have found that speed eating correlates to obesity risk [10,11,12,38]. Hawton et al. demonstrated that slower eating might affect fullness, appetite and have a role in hormonal pathways [39]. The “eating speed” mechanism has a strong relation with mastication, which may also affect energy intake and body weight, increasing the food surface to facilitate digestion, satiety and hormone response. Nevertheless, the role of digestion and satiety hormones on eating speed has not yet been determined. With the above approaches in mind, an increasing number of studies have examined the interaction between eating speed and metabolic syndrome (MetS), diabetes, non-alcoholic fatty liver disease and cardiovascular diseases [28,40,41,42]. All findings have demonstrated a positive relationship between speed eating and these diseases and pointed to the need to analyse these in connection to obesity.

Obesity is a widely growing problem that needs to be monitored, and early markers of its appearance need to be determined. The classical definition of obesity covers body fat accumulation and anthropometrical indicators such as body mass index, waist circumference, waist–hip ratio and waist–height ratio. They are commonly used to determine its severity and have frequently been included in cohort studies investigating health risk [43,44,45,46]. Those indicators reflect the effects of long-term behaviour on the body [47,48]. In contrast, eating speed might be an early predictor of long-term dietary behaviours connected to social and cultural influences. Interestingly, eating speed has been widely used as a part of cohort studies in Japan to determine the risk factors for obesity [28,49,50,51]. Nevertheless, eating speed is still a missing component of nutrition studies in non-Asian countries, where new modifiable obesity risk markers are still being sought, and their use might reduce the risk of its occurrence. 

Therefore, it is necessary to evaluate how fast or slow eating as a form of behaviour affects obesity. The interaction between weight gain and eating speed may be a part of dietary consultations and is beneficial for obesity prevention. 

It is crucial to increase knowledge of the potential influence of eating speed on noncommunicable diseases, predominantly obesity and its indicators. Although awareness of eating speed has increased, there are no literature reviews examining the relationship between eating speed and anthropometric indicators of obesity, such as BMI and waist circumference. This may be due to insufficient attention to the importance of eating speed in developing and protecting obesity and the lack of reviews evaluating existing studies. 

Therefore, this review seeks to address the relationship between eating speed and obesity indicators and revises recent studies involving eating speed on obesity in the general population and subjects with noncommunicable diseases.

## 2. Materials and Methods

The protocol was registered in the “PROSPERO International prospective register of systematic reviews” number: PROSPERO 2021 CRD42021224322.

### 2.1. Study Selection Process

Studies were selected to identify and define the influence of eating speed on obesity. A systematic search of the literature was performed in the following databases: Web of Science (Clarivate Analytics, PA, USA) (https://www.webofknowledge.com, accessed on 16 January 2021), PubMed (National Institute of Health, MA, USA) (https://www.ncbi.nlm.nih.gov/pubmed, accessed on 16 December 2020), Scopus (Elsevier, RELX Group plc, London, UK), (https://www.scopus.com, accessed on 10 February 2021) and Science Direct (Elsevier, RELX Group plc, London, England, UK) (https://www.sciencedirect.com/, accessed on 14 January 2021). The strategy was based upon the following index terms, titles or abstracts: (eating speed OR speed eating OR fast-eating OR slow eating OR quick eating) AND (obesity OR Body mass index OR waist circumference OR waist-hip ratio OR waist height ratio OR fat accumulation OR body fat percentage OR metabolic syndrome) AND (Human).

**Figure 1:**
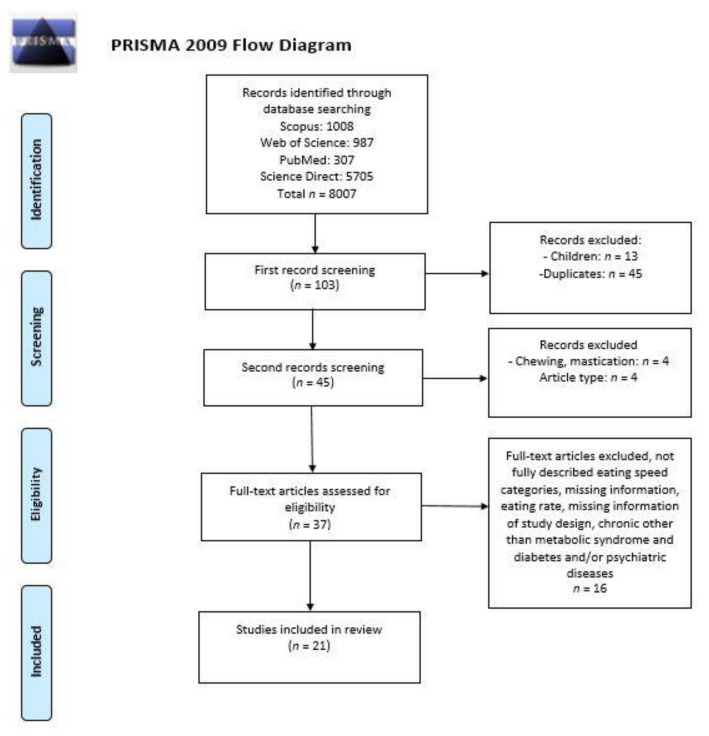
Procedure of identification and screening process for selecting reviewed articles exploring the relationship between eating speed and obesity indicators in adults.

Figure 1 represents the identification and screening process for selecting reviewed articles using the PRISMA (preferred reporting items for systematic reviews) diagram. The study selection process (Figure 1) was based on assessing the article’ titles, abstracts and full texts by two independent researchers for each database. At each step, all disagreements between the researchers were resolved in consultation with the review coordinator. Papers qualified during the title assessment process were included in the next step. Full-texts of all records selected in the first step were searched by using the Poznan University of Life Sciences Library.

### 2.2. Inclusion and Exclusion Criteria

Only original articles in the English language were included in this revision. Studies selected involved adults and both sexes, with no age or weight status limitations. Exclusion criteria were studies on pregnancy, lactation and psychological disorders (eating disorders or involved psychology therapy). Studies were excluded if: (1) they were not an original research paper (i.e., short communication, review, meta-analysis); (2) they had no clear information for the measurement of anthropometrical parameters; (3) eating speed was not analysed with obesity indicators; (4) the subjects had any psychiatric disease or eating disorders; (5) they investigated eating rate instead of eating speed. All studies assessed the relation of anthropometric parameters of obesity (e.g., BMI, waist circumference, waist-hip-ration, waist-height ratio and body composition), with eating speed (e.g., slow, fast). The search included both qualitative and quantitative studies. 

### 2.3. Data Analysis

**Figure 2:**
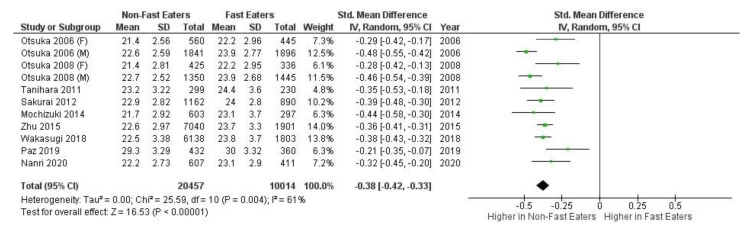
Forest plot of meta-analysis slow and fast eating speed according to the included observational studies.

In the 21 studies examined in the review study, eight were not included in the meta-analysis due to a lack of statistical data such as percentage information of BMI categories and median values. Five studies were not included in the meta-analysis review due to too low or too high sample size or too few or too many subjects. According to Mettelli and Chaimani, significant heterogeneity in the sample size of observational studies included in the meta-analysis creates high bias. The variety of the categories of eating speed and difference in study design are required to perform sub-analyses of each group separately. Therefore, the studies that used more than two categories were combined from “very slow”, “slow” and “medium/moderate” categories into “non-fast”. Similarly, studies with “fast” and “very fast” categories were combined into “fast” in the meta-analysis [52]. After quality evaluation, eight studies remained for the meta-analysis and two studies were analysed with the two subgroups female and male. The meta-analysis was conducted using Review Manager (RevMan) software 5.4 (Copenhagen: The Nordic Cochrane Centre, The Cochrane Collaboration, 2014). A meta-analysis was conducted with these eight studies with gender separation in two of them, as shown in Figure 2. The magnitude of heterogeneity is known as low (I2 = 0 to 24%), moderate (I2 = 25 to 49%), large (I2 = 50 to 74%) or extreme (I2 = 75 to 100%) heterogeneity. Studies were analysed to compare non-fast and fast eaters when the heterogeneity of the studies was evaluated as over 50% by the I2 statistic. A *p*-value of ≤0.05 was considered statistically significant.

### 2.4. Quality Assessment

A quality assessment of articles was performed with a MINORS (methodological index for non-randomised studies) checklist and presented in Supplementary Table S1 [52]. The included studies’ overall quality was low due to methodological inconsistencies and heterogeneity in terms of statistical and clinical characteristics.

## 3. Results

**Figure 3:**
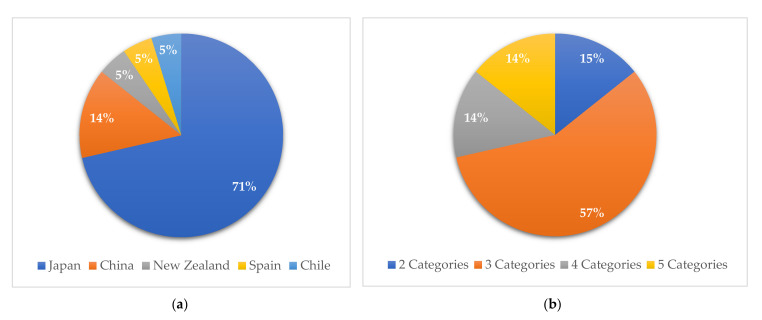
(**a**) Percentile distribution of the reviewed articles by country; (**b**) eating speed categories in the reviewed articles by percentage.

A total of 4523 studies in four different databases were scanned for inclusion in this systematic review. After the elimination process (Figure 1), a total of six [42,50,51,52,53,54] longitudinal studies and fifteen [43,44,45,46,48,55,56,57,58,59,60,61,62,63,64] cross-sectional studies were included. Eighteen articles [42,43,44,45,46,50,51,52,55,56,57,58,59,60,61,62,63,64] studied the general population, while three [48,53,54] included subjects with noncommunicable diseases, such as metabolic syndrome and diabetes. Figure 3a illustrates that the majority of the reviewed articles were from Asian countries, i.e., 71% from Japan (fifteen studies) [42,43,44,45,50,51,52,53,54,55,58,59,61,63,64] and 16% from China (three studies) [48,57,62]. Figure 3b shows the distribution of research by eating speed categories. Table 1 shows the details of the categories of each level. Most studies have used three categories in different variations: slow or slowly, normal, moderate or medium and fast or quickly. Five studies [51,52,57,61,64] combined categories after data collection due to the low variability of the final responses (Table 1).

A meta-analysis was completed on five comparable studies. Figure 2 presents the forest plot, and the overall effect size between slow eating speed and fast eating speed group was −1.44 (95% CI = −5.49 to −0.24, I2 100%, *p* < 0.001). 

The existence of a relationship between eating speed and indicators was marked in the tables. Table 2 summarises the design and results from articles on the relationship between eating speed and obesity indicators in the general population. Table 2 presents eighteen studies conducted in the general population, of which fourteen are cross-sectional studies [26,28,40,54,57,58,59,60,61,62,63], and four are longitudinal studies [25,51,53,55]. All articles presented in Table 2 found a significant relationship between eating speed and BMI. In addition, seven studies [25,26,51,57,58,59,60] concluded an association between WC and ES in the general population, while one study found no relation between them [40]. Table 3 provides a summary of design and results from articles performing ES and obesity indicators in subjects with noncommunicable diseases. Table 3 shows three studies, including two longitudinal [49,56] and one cross-sectional [42]. One of the studies shown was conducted on people with diabetes [56], one compared people with and without diabetes [49] and one compared people with or without metabolic syndrome [42]. In all three studies performed on people with noncommunicable diseases, there was a significant association between ES and both BMI and WC [42,49,56]. Of the twenty-one studies that examined eating speed and obesity indicators, eight investigated gender differences in the results [25,40,42,49,51,54,56,57]. Brief results from these studies are presented in the additional information section in Table 2.

## 4. Discussion

The current review summarised cross-sectional and longitudinal articles to investigate the relation between obesity indicators and ‘eating speed’ as a dietary behaviour. In this review, obesity indicators were BMI and waist circumference. Collectively, these data support previous studies that eating speed is significantly related to obesity and obesity indicators.

### 4.1. The Correlation between Eating Speed and Obesity

In almost all of the articles scanned, an association between BMI and eating speed in the general population along with subjects with noncommunicable diseases was noted. The results from the meta-analysis and systematic reviews are discussed together. Studies with both healthy or noncommunicable disease samples found a relationship between obesity and eating speed, except for one study involving subjects aged 55–80 [40]. Although BMI is a simple tool used worldwide to classify obesity and is accepted as an accurate measurement in general populations, it is known that it has limitations to assess central obesity and fat distribution [66]. Therefore, waist circumference and other obesity indicators such as body fat percentage and visceral fat distribution were included in this review. Eleven of the reviewed articles examined the relationship between waist circumference and eating speed in addition to BMI. Almost all articles found an association between eating speed and waist circumference [25,26,42,49,51,56,57,58,59,60].

In contrast, a cross-sectional study conducted in Spain found no significant association between ES and WC and BMI [40]. A study compared objective and subjective ES and examined its relationship to body composition. It showed that slow eaters had a lower fat percentage than those in the other categories [24]. Another study on the correlation between body composition and eating speed found that fast eaters had greater visceral fat distribution than slower eaters [57]. Physical activity is one of the possible confounding variables which might significantly influence body weight [67]. Some of the studies included in the review examined the relationship between eating speed and obesity after physical activity adjustment. The results support the view that obesity is associated with eating speed, regardless of physical activity level [26,53,65]. However, further studies need to be conducted to investigate eating speed as an independent factor in weight gain and reduction and analyse physical activity changes as an independent variable influencing a speed-eating modification to support weight management and pro-healthy behaviours.

Nevertheless, all of the mentioned findings seem consistent with supporting the relationship between obesity indicators and eating speed. After all, the design of the observational studies included in this review does not make it possible to distinguish this relation’s direction. It is unclear whether faster eating contributes to a higher BMI or if subjects with a higher BMI tend to eat faster than those with a lower BMI.

However, longitudinal studies included in this review determined the long-term effect of ES on obesity. A 6-year longitudinal study investigated the effect of eating speed changes on obesity and BMI in a patient with diabetes and found that lowering eating speed reduces obesity and its indicators [56]. These results were consistent with an 8-year retrospective study which reported that fast eaters gained more weight than slow or medium eaters among Japanese middle-aged male subjects [53]. These data suggest that ES is one of the vital dietary behaviours in weight control, and slowing down ES may contribute to obesity prevention. However, more studies are needed to investigate the effect of slowing ES for obesity prevention and clarify methods and efficient strategies to decrease meal intake speed.

### 4.2. Sex Difference in Eating Speed

Prior studies have noted the importance of sex as a factor affecting dietary behaviours and obesity [68]. The sex difference in eating speed was investigated in eight studies [25,40,42,49,51,54,56,57]. The results from six studies showed that fast eaters were more likely to be male, or that there was a significant relationship between gender and ES categories [25,42,49,51,56,57]. In contrast, another study reported that fast eaters were most frequently younger women who had higher BMI than slower eaters [40]. Additionally, one study found no relation between sex and eating speed [54]. The sex difference in eating physiology is well known [34], and this might be the reason for the sex difference in eating speed. There are still many unanswered questions about whether the main cause of this difference is physiologically based or lifestyle-based.

### 4.3. The Relation between Eating Speed and Other Noncommunicable Diseases

The increased prevalence of noncommunicable diseases, such as diabetes, cardiovascular diseases, non-alcoholic fatty liver diseases, constitute a significant public health risk and lead to increased morbidity and mortality [69]. The strong interaction between dietary behaviours and noncommunicable diseases has been highlighted in the literature [69,70]. Three studies investigating the relationship between metabolic syndrome and ES were included in the review [25,26]. Zhu et al. reported that metabolic syndrome incidence was correlated with eating speed even after age and sex adjustment [25]. Another study in which age and multiple adjustments (including physical activity) were made had supportive results [26]. Those findings were supported by a recent follow-up study that indicated that fast eating speed was associated with an increased likelihood of developing metabolic syndrome independently of total energy intake [51]. Similarly, another study noted that participants who ate faster were more likely to have cardiovascular risk factors such as high triglycerides, low HDL and high blood pressure [42]. Very little was found in the literature on the effect of eating speed on glucose control, an essential parameter for diabetes risk and prevention. Iwasaki et al. performed a follow-up study to investigate the effect of ES on glucose control in Japanese adults and reported that fast eaters are more likely to have poor glycaemic control than slow-medium eaters after age-adjusted [55]. Another study focused on diabetes risk and ES and indicated that fast eaters were more likely to have diabetes than slow eaters. Those authors underlined that fast eating could be a risk factor for diabetes [49]. In partial contrast with the previously mentioned two studies, Sakurai et al. reported that the association between fast eating and diabetes incidence disappeared after the adjusted BMI analysis [28]. These findings need to interpret body weight and obesity to diabetes risk and eating speed. 

### 4.4. Strengths and Limitations

The article has several limitations. First of all, the analysed studies examined eating speed at different speed ranges from 2 to 5. Twelve out of nineteen studies evaluated eating speed by dividing it into three categories: slow, medium and fast (Table 1). The lack of standardisation in categories may confuse the data’s evaluation and create difficulties in comparing different studies results. The conducted meta-analysis had a large level of heterogeneity across the studies. The included studies’ overall quality was evaluated and it was noted that there was the limitation of longitudinal studies, methodological inconsistencies and heterogeneity in terms of statistical characteristics [62]. Another weakness of the reviewed articles is the validity of the self-reported eating speed question. To validate the self-reported questionnaire, most researchers in their studies compared self-reported eating speed with the feedback from a participant’s friend [71].

In contrast to many studies in the literature, a study conducted in Korea used self-reported eating duration instead of eating speed categories [72]. Standard methods and categories should be established in the evaluation of eating speed. Three studies also used self-reported weight and height for the calculation of BMI [53,54,73]. Although self-reported anthropometric data are commonly used, people tend to overestimate their height and underestimate their weight and make errors in other parts of questionnaires such as gender and ethnicity, which are necessary to take into account [74,75,76]. It is also necessary to emphasise that a lack of information for physical activity level presented in the examined studies may cause bias when evaluating the results.

Another point that should be emphasised is that the reviewed articles were mainly from Asian countries. Three non-Asian countries were included in the review: New Zealand, Chile and Spain. It should be underlined that eating culture and environmental factors may have an influence on dietary behaviours as well as eating speed. This study’s strength is that it is the first systematic review study to examine the relationship between anthropometric indicators of obesity and eating speed. Eating speed, a dietary behaviour that is simple to assess, can be added to dietary surveys and used in clinical practices focused on weight control.

## 5. Conclusions

In conclusion, the analysed studies and data indicate that eating speed is associated with obesity indicators, most strongly with BMI. The findings reported here shed new light on the possible role of eating speed in preventing noncommunicable diseases such as diabetes and cardiovascular disease. However, interventional studies are required to investigate the causation for the relationship between eating speed and obesity. Further studies should perform advanced nutritional assessment techniques to determine the effect of eating speed on obesity and examine whether eating speed influences body fat percentage and distribution. Moreover, to better determine eating speed, a validated questionnaire with standard speed categories should be established. Determining and slowing down the eating speed could be an efficient strategy for the weight management part of dietary counselling. Therefore, considerably more national and cross-national studies will need to be done to determine the effect of ethnicity, sex, food culture and health status on eating speed.

## Figures and Tables

**Table 1:** Eating speed categories of the reviewed articles.

Study	Sample	Assessment Tool	Eating Speed Categories
Two categories of studies			
[25] Japan 2015	*n* = 8941 adults	Self-reported	Not Fast, Fast
[53] Japan 2011	*n* = 529 male workers	Self-reported	Fast, (Medium and Slow) (Three categories planned initially)
[54] Chile 2013	*n* = 292 adults	Self-reported	Slow, Fast
Three categories of studies			
[55] Japan 2019	*n* = 5479 adults	Self-reported	Slowly, Medium, Quickly
[40] Spain 2019	*n* = 792 adults	Self-reported	Slow, Medium, Fast
[42] China 2018	*n* = 7972 adults with MetS and without MetS	Self-reported	Slow, Medium, Fast
[56] Japan 2018	*n* = 59,717 adults with type 2 diabetes	Self-reported	Slow, Normal, Fast
[49] Japan 2019	*n* = 197,825 adults with diabetes, without diabetes	Self-reported	Slow, Moderate, Fast
[26] Japan 2014	*n* = 56,865 adults	Self-reported	Slow, Normal, Fast
[57] Japan 2019	*n* = 381 non-obese adults	Self-reported	Slowly, Medium, Quickly
[58] Japan 2014	*n* = 900 healthy women	Self-reported	Slow (Very Slow and Relatively Slow), Medium, Fast (Relatively Fast and Very Fast) (Five categories planned initially)
[27] Japan 2018	*n* = 7941 adults	Self-reported	Slow, Normal, Fast
[28] Japan 2012	*n* = 2050 middle-aged men	Self-reported	Slow, Medium, Fast
[59] Japan 2017	*n* = 84 female college students	Self-reported	Slow, Moderate, Fast
[51] Japan 2020	*n* = 1018 adults	Self-reported	Slow (Very Slow and Relatively Slow), Medium, Fast (Relatively Fast and Very Fast (Five categories planned initially)
Four categories of studies			
[60] Japan 2018	*n* = 863 adult working men	Self-reported	(Slow and very Slow), Ordinary, Fast, Very fast (Five categories planned initially)
[61] China 2019	*n* = 536 college students.	Self-reported	Slow, Normal, Slightly Fast, Fast.
[62] China 2019	*n* = 536 undergraduates	Self-reported	Slow (Very Slow and Slow), Ordinary, Fast, Very Fast (Five categories planned initially)
Five categories of studies			
[63] New Zealand 2011	*n* = 1515 middle-age women	Self-reported	Very Slow, Relatively Slow, Medium, Relatively Fast, Very Fast
[64] Japan 2007	*n* = 3465 non-diabetic workers	Self-reported	Very slow, Relatively slow, Medium, Relatively fast, Very fast
[65] Japan 2006	*n* = 4742; men = 3737; women = 1005	Self-reported	Very Slow, Relatively Slow, Medium, Relatively Fast, Very Fast

**Table 2:** Summary of the design and results from articles investigating the relationship between eating speed and obesity indicators in the general population.

Study/Country	Study Design	Methods	Participants	Age (year)	Obesity Indicators	Eating Speed	Outcome		Additional Information
							BMI	WC	
[63] (Leong, Madden, Gray, Waters, & Horwath, 2011) New Zealand	Cross-sectional study	Self-reported eating speed and BMI	*n* = 1515 middle-age women	45.5 ± 3.2	BMI: 25.8 ± 1.2 kg/m^2^	Five categories: Very slow, Relatively slow, Medium, Relatively fast, Very fast	+	NA	BMI was significantly associated with eating speed both in unadjusted and after adjusting for age, ethnicity, socioeconomic status and physical activity.
[55] (Iwasaki, Hirose, Azuma, Ohashi, et al., 2019) Japan	Cohort study	Anthropometric measurement for BMI. Self-reported eating speed	*n* = 5479 adults	49 (45–54) ^¥^	BMI: 22.4 (20.4–24.6) ^¥^ kg/m^2^	Three categories: Slowly, Medium, Quickly	+	NA	Quick eaters were significantly more likely to be male.
[40] (Paz-Graniel, Babio, Mendez, & Salas-Salvadó, 2019) Spain	Cross-Sectional Study	Anthropometric measurement for BMI and WC. Self-reported eating speed	*n* = 792 adults	67.5 ± 5.86	BMI: 29.62 ± 3.32 kg/m^2^ WC Men: Slow: 102.74 ± 9.03 cm Medium: 103.37 ± 8.45 cm Fast: 103.24 ± 8.41 cm Women Slow: 99.23 9.6 ± 5 cm Medium: 98.27 ± 7.80 cm Fast: 99.79 ± 8.56 cm	Three categories: Slow, Medium, Fast	-	-	Fast eaters were most frequently younger women who had higher BMI than slower eaters.
[25](Zhu, Haruyama, Muto, & Yamazaki, 2015) Japan	Follow-up Cohort study	Anthropometric measurement for BMI and WC. Self-reported eating speed.	*n* = 8941 adults	63.7 ± 7.9	BMI: 22.8 ± 3.1 kg/m^2^. WC: 82 ± 8.8 cm.	Two categories Not fast, fast	+	+	In an age- and sex-adjusted analysis, eating speed was significantly associated with the incidence of metabolic syndrome.
[60] (Sonoda et al., 2018) Japan	Cross-sectional study	Anthropometric measurement for BMI and WC. Self-reported eating speed.	*n* = 863 adult working men	>39, 40–44, 45–49, ≤50	BMI: (Slow, very slow): 24.0 ± 3.3 kg/m^2^, Ordinary: 24.9 ± 3.8, fast: 26.1 ± 3.8, very fast: 27.0 ± 3.3. WC: (slow, very slow): 82.4 ± 8.0, ordinary: 85.3 ± 10.1 fast: 87.9 ± 9.9, Very fast: 89.0 ± 8.1.	Four categories: (Slow and very Slow), Ordinary, Fast, Very fast	+	+	There were significant differences in BMI and waist circumference between slow eaters and fast eaters in some age groups.
[61] (Xie et al., 2019) China	Cross-sectional study	Anthropometric measurement for BMI. Self-reported eating speed.	*n* = 536 college students. Male = 257, Female = 279.	Male: 22.07 ± 3.42, Female: 21.10 ± 2.73	BMI: Underweight: 17.61 ± 0.76 kg/m^2^, normal weight: 21.14 ± 1.69 kg/m^2^, Overweight: 27.48 ± 2.19 kg/m^2^.	Four categories: Slow, Normal, Slightly fast, Fast.	+	NA	
[53] (Tanihara et al., 2011) Japan	Retrospective longitudinal study	Self-reported eating speed and BMI.	*n* = 529 male workers	4 categories: 20–29, 30–39, 40–49, 50–59	BMI: 23.7 ± 3.4 kg/m^2^.	Two categories: Fast, (Medium and Slow)	+	NA	In both baseline and follow-up studies, BMI and weight were related to eating speed.
[26] (Nagahama et al., 2014) Japan	Cross-sectional study	Anthropometric measurement for BMI and WC. Self-reported eating speed.	*n* = 56,865 men = 41,820, Women = 15,045	Age Men: Slow 46.9 ± 12.3 Normal 46.9 ± 10.9 Fast 45.0 ± 10.4 Women Slow 43.5 ± 12.5 normal 47.2 ± 11.6 fast 46.7 ± 11.2	BMI Men: Slow: 22.4 ± 3.3 kg/m^2^. Normal: 23.4 ± 3.3 kg/m^2^. Fast: 24.6 ± 3.7 kg/m^2^. Women; Slow: 21.0 ± 3.5 kg/m^2^. Normal: 21.8 ± 3.5 kg/m^2^. Fast: 22.5 ± 3.8 kg/m^2^. WC: Men: Slow: 80.3 ± 9.2 cm. Normal: 82.9 ± 9.0 cm. Fast: 86.0 ± 9.8 cm. Women: Slow: 75.5 ± 9.5 cm. Normal: 77.7 ± 9.4 cm. Fast: 79.6 ± 9.8 cm.	Three categories: Slow, Normal, Fast	+	+	Fast eaters were more likely to have central obesity compared to slow eaters.
[57] (Iwasaki, Hirose, Azuma, Watanabe, et al., 2019) Japan	Cross-sectional study	Anthropometric measurement for BMI and WC. VFA and SFA measured by CT. Self-reported eating speed.	*n* = 381 non-obese adults	53 (45, 59) ^¥^	BMI: 23.2 (21.4, 25.4) ^¥^ kg/m^2^ WC: 81 (76, 86) ^¥^ cm VFA: 98 (59, 140) ^¥^ cm^2^. SFA: 136 (101, 174) ^¥^ cm^2^.	Three categories: Slowly, Medium, Quickly	+	+	Eating speed was significantly associated with VFA, but not with SFA.
[58] (Mochizuki et al., 2014) Japan	Cross-sectional study	Anthropometric measurement for BMI and WC. Self-reported eating speed.	*n* = 900 healthy women	53.1 ± 7.1	BMI = 22.2 ± 3.2 kg/m^2^ WC = 77.0 ± 9.7 cm	Three categories: (Very Slow and Relatively Slow), Medium, (Relatively Fast and Very Fast)	+	+	
[27] (Wakasugi, Kazama, & Narita, 2018) Japan	Cross-sectional study	Anthropometric measurement for BMI. Self-reported eating speed.	*n* = 7941 adults	66.9 1 ± 3.9	BMI = 22.8 ± 3.5 kg/m^2^.	Three categories: Slow, Normal, Fast	+	NA	
[28] (Sakurai et al., 2012) Japan	Cross-sectional study	Anthropometric measurement for BMI. Self-reported eating speed.	*n* = 2050 middle aged men	45.9 ± 6.0	BMI = 23.4 ± 2.9 kg/m^2^.	Three categories: Slow, medium, fast	+	NA	After adjusting for age, eating speed was associated with obesity risk.
[62] (Shan et al., 2019) China	Cross-sectional study	Anthropometric measurement for BMI. Self-reported eating speed.	*n* = 536 undergraduates	20(17–22) ^¥^	BMI Categories: Underweight: 12.5% Normal: 73.9% Overweight and obese: 13.6%	Four categories: (Very slow and slow), Ordinary, Fast, Very fast	+	NA	Eating very fast was positively associated with overweight and obesity.
[59] (Hamada et al., 2017) Japan	Cross-sectional study	Anthropometric measurement for BMI, WC and BF%. Abdomen and Hip circumferences. Self-reported eating speed.	*n* = 84 female college students.	19 ± 1	BMI: 22 ± 3 kg/m^2^. BF%: 27 ± 4 WC = 69 ± 7 cm. Hip circumferences: 93 ± 6 cm.	Three categories: Fast, moderate, slow	+	+	The objective eating speed measurement was performed and had a similar result as subjective eating speed.
[54] (Oda-Montecinos, Saldaña, & Andrés, 2013) Chile	Cross-sectional study	Self-reported eating speed and BMI.	*n* = 292 adults	38.3 ± 11.76	BMI: 26.58 ± 4.39 kg/m^2^	Two categories: Slow, Fast	+	NA	Fast eating was significantly different between normal weight and overweight subjects. There was no difference between genders.
[51] (Nanri et al., 2020) Japan	Follow-up study	Anthropometric measurements for BMI and WC. Self-reported eating speed	*n* = 1018	Slow: 42.6 ± 9.7 Medium: 43.3 ± 8.2 Fast: 41.1 ± 7.9	BMI: Slow: 21.7 ± 2.8 kg/m^2^ Medium: 22.4 ± 2.7 kg/m^2^ Fast: 23.1 ± 2.9 kg/m2 WC: Slow: 77.7 ± 7.0 cm Medium: 79.4 ± 7.4 cm Fast: 81.8 ± 8.1 cm	Three Categories: Slow (Very Slow and Relatively Slow), Medium, Fast (Relatively Fast and Very Fast)	+	+	Eating speed was related to BMI change during a three-year follow-up study.
[64] (Otsuka et al., 2008)	Cross-sectional	Anthropometric measurements for BMI. Self-reported eating speed	*n* = 3465 non-diabetic workers	Men: 48.2 ± 7.1 Women: 46.3 ± 6.9	BMI: Men: 23.3 ± 2.6 kg/m^2^ Women: 21.8 ± 2.7 kg/m^2^	Five categories: Very slow, Relatively slow, Medium, Relatively fast, Very fast	+	NA	Eating speed was positively related to energy intake in both sexes.
[65] (Otsuka et al., 2006)	Cross-sectional	Anthropometric measurements for BMI. Self-reported eating speed	*n* = 4742 men = 3737 women = 1005	Men: 48.2 ± 7.1 Women: 46.3 ± 7	BMI: Men: 23.3 ± 2.7 kg/m^2^ Women: 21.8 ± 2.8 kg/m^2^	Five categories: Very Slow, Relatively Slow, Medium, Relatively Fast, Very Fast	+	NA	

“^¥^” presents Median (Q1–Q3). “+” presents a statistically significant relation, “-” presents a statistically insignificant relation, NA: Not Applicable, BMI: Body Mass Index, WC: Waist Circumference, VFA: Visceral Fat Area, SFA: Subcutaneous Fat Area.

**Table 3:** Summary of the design and results from articles analysing the relation between eating speed and obesity indicators in subjects with noncommunicable diseases.

Study/Country	Study Design	Methods	Participants	Age (year)	Obesity Indicators	Eating Speed	Outcome		Additional Information
							BMI	WC	
[42] (Tao et al., 2018) China	Cross-sectional study	Anthropometric measurement for BMI and WC. Self-reported eating speed.	*n* = 7972 adults With MetS and without MetS	38 (31–48) ^¥^	BMI ^¥^: Male Slow: 24.2 (21.8–26.8) kg/m^2^, Medium: 25.0 (23.1–27.0) kg/m^2^, Fast: 25.7 (23.5–28.1) kg/m^2^. Female Slow: 21.1 (19.1–23.1) kg/m^2^, Medium: 21.8 (20.2–24.1) kg/m^2^, Fast: 22.5 (20.524.8) kg/m^2^, WC ^¥^: Male Slow: 84 (78–92)cm, Medium: 87 (81–93)cm, Fast: 88 (83–95) Female Slow: 70 (65–76)cm, Medium: 72 (68–78)cm Fast: 73 (68–79) cm.	Three categories: Slow, Medium, Fast	+	+	Eating speed was significantly related to excessive salt intake in both genders but not related to excessive sugar intake in both genders.
[56] (Hurst & Fukuda, 2018) Japan	Longitudinal study	Anthropometric measurement for BMI and WC. Self-reported eating speed.	*n* = 59,717 adults with type 2 diabetes	40–69 Slow: 46.5 ± 11.7, Normal: 48.1 ± 10.6, Fast: 46.6 ± 10.4.	BMI: slow: 22.3 ± 4.0 kg/m^2^, normal: 23.4 ± 3.9 kg/m^2^, Fast: 25.0 ± 4.4 kg/m^2^. WC Slow: 80.1 ± 10.6 cm, Normal: 82.8 ± 10.4 cm. Fast: 86.8 ± 11.1 cm.	Three categories: Slow, Normal, Fast	+	+	Lowering the eating speed was related to the reduction of BMI and WC.
[49] (Kudo et al., 2019) Japan	Cohort study	Anthropometric measurement for BMI and WC Self-reported eating speed.	*n* = 197,825 adults With diabetes, without diabetes	Age 63.7 ± 7.7	BMI 22.9 ± 3.1 kg/m^2^, WC 83.2 ± 8.8 cm	Three categories: Slow, Moderate, Fast Subcategories: Non-fast (Slow and Moderate), Fast	+	+	After adjusting multiple factors (age, weight, blood pressure, etc.), fast eating speed was significantly related to developing diabetes.

“^¥^” presents Median (Q1–Q3). “+” presents a statistically significant relation, “-” presents a statistically insignificant relation, NA: Not Applicable, BMI: Body Mass Index, WC: Waist Circumference, VFA: Visceral Fat Area, SFA: Subcutaneous Fat Area.

## Data Availability

Additional data are available upon request to the author for correspondence.

## Supplementary Materials

The following are available online at https://www.mdpi.com/article/10.3390/healthcare9111559/s1, Supplementary Table S1: Quality assessment of the articles.
