# Supplementary material for: Self-Reported Eating Speed Is Associated with Indicators of Obesity in Adults: A Systematic Review and Meta-Analysis
Source: Healthcare (Basel). 2021 Nov 16;9(11):1559. doi: 10.3390/healthcare9111559 (PMC8619990; doi:10.3390/healthcare9111559)
Supplement: Supplementary file 1 [file healthcare-09-01559-s001.zip › healthcare-1415316-supplementary.pdf]

Supplementary Table S1 Quality assessment of the articles

	Methodological item for non-randomized studies score (MINORS)								Additional criteria in the case of comparative study in MINORS				
Study	1.A clear stated aim	2.Inclusion of consecutive patients	3.Prospective collection of data	4.Endpoints appropriate to the aim of the study	5.Unbiased assessment of the study endpoint	6.Follow-up period appropriate to the aim of the study	7.Loss to follow up less than 5%	8.Prospective calculation of the study size	9.An adequate control group	10.Contemporary groups	11.Baseline equivalence of groups	12.Adequate statistical analyses	Total
[50] (Leong, Madden, Gray, Waters, & Horwath, 2011) New Zealand	2	1	2	2	1	NA	NA	NA	NA	NA	NA	NA	8
[44] (Iwasaki, Hirose, Azuma, Ohashi, et al., 2019) Japan	2	1	2	2	1	2	0	0	NA	NA	NA	NA	10
[39] (Paz-Graniel, Babio, Mendez, & Salas-Salvadó, 2019) Spain	2	2	2	2	1	NA	NA	NA	NA	NA	NA	NA	9
[45](Zhu, Haruyama, Muto, &	2	2	2	2	1	1	2	1	NA	NA	NA	NA	13

Yamazaki, 2015) Japan													
[51] (Sonoda et al., 2018) Japan	2	1	1	2	1	NA	NA	NA	NA	NA	NA	NA	7
[52] (Xie et al., 2019) China	2	2	2	2	1	NA	NA	NA	NA	NA	NA	NA	9
[46] (Tanihara et al., 2011) Japan	1	1	2	2	1	2	0	0	NA	NA	NA	NA	9
[53](Nagahama et al., 2014) Japan	2	2	2	2	1	NA	NA	NA	NA	NA	NA	NA	9
[54] (Iwasaki, Hirose, Azuma, Watanabe, et al., 2019) Japan	2	1	2	2	1	NA	NA	NA	NA	NA	NA	NA	8
[55] (Mochizuki et al., 2014) Japan	2	2	2	2	1	NA	NA	NA	NA	NA	NA	NA	9
[56] (Wakasugi, Kazama, & Narita, 2018) Japan	2	2	2	2	1	NA	NA	NA	NA	NA	NA	NA	9
[62] (Sakurai et al., 2012) Japan	2	2	2	2	1	2	2	2	NA	NA	NA	NA	15
[59] (Shan et al., 2019) China	2	1	1	2	1	NA	NA	NA	NA	NA	NA	NA	7

[57] (Hamada et al., 2017) Japan	2	2	2	2	1	NA	NA	NA	NA	NA	NA	NA	9
[58] (Oda-Montecinos, Saldaña, & Andrés, 2013) Chile	2	1	2	1	1	NA	NA	NA	NA	NA	NA	NA	7
[47] (Nanri et al., 2020) Japan	2	2	2	2	1	2	0	0	NA	NA	NA	NA	11
[60] (Otsuka et al., 2008)	2	2	2	2	1	NA	NA	NA	NA	NA	NA	NA	9
[61] (Otsuka et al., 2006)	2	2	2	2	1	NA	NA	NA	NA	NA	NA	NA	9
[41] (Tao et al., 2018) China	2	2	2	2	2	NA	NA	NA	NA	NA	NA	NA	10
[48] (Hurst & Fukuda, 2018) Japan	2	1	2	2	1	2	2	0	NA	NA	NA	NA	12
[49] (Kudo et al., 2019) Japan	2	2	1	2	1	2	0	0	NA	NA	NA	NA	10

NA: Not Applicable.
